# Nanoparticle Incorporation of Melittin Reduces Sperm and Vaginal Epithelium Cytotoxicity

**DOI:** 10.1371/journal.pone.0095411

**Published:** 2014-04-18

**Authors:** Andrew P. Jallouk, Kelle H. Moley, Kenan Omurtag, Grace Hu, Gregory M. Lanza, Samuel A. Wickline, Joshua L. Hood

**Affiliations:** 1 Department of Medicine, Division of Cardiology, Consortium for Translational Research in Advanced Imaging and Nanomedicine, Washington University School of Medicine, St. Louis, Missouri, United States of America; 2 Department of Obstetrics and Gynecology, Washington University School of Medicine, St. Louis, Missouri, United States of America; Institute of Human Virology, Baltimore, United States of America

## Abstract

Melittin is a cytolytic peptide component of bee venom which rapidly integrates into lipid bilayers and forms pores resulting in osmotic lysis. While the therapeutic utility of free melittin is limited by its cytotoxicity, incorporation of melittin into the lipid shell of a perfluorocarbon nanoparticle has been shown to reduce its toxicity *in vivo*. Our group has previously demonstrated that perfluorocarbon nanoparticles containing melittin at concentrations <10 µM inhibit HIV infectivity *in vitro*. In the current study, we assessed the impact of blank and melittin-containing perfluorocarbon nanoparticles on sperm motility and the viability of both sperm and vaginal epithelial cells. We found that free melittin was toxic to sperm and vaginal epithelium at concentrations greater than 2 µM (p<0.001). However, melittin nanoparticles were not cytotoxic to sperm (p = 0.42) or vaginal epithelium (p = 0.48) at an equivalent melittin concentration of 10 µM. Thus, nanoparticle formulation of melittin reduced melittin cytotoxicity fivefold and prevented melittin toxicity at concentrations previously shown to inhibit HIV infectivity. Melittin nanoparticles were toxic to vaginal epithelium at equivalent melittin concentrations ≥20 µM (p<0.001) and were toxic to sperm at equivalent melittin concentrations ≥40 µM (p<0.001). Sperm cytotoxicity was enhanced by targeting of the nanoparticles to the sperm surface antigen sperm adhesion molecule 1. While further testing is needed to determine the extent of cytotoxicity in a more physiologically relevant model system, these results suggest that melittin-containing nanoparticles could form the basis of a virucide that is not toxic to sperm and vaginal epithelium. This virucide would be beneficial for HIV serodiscordant couples seeking to achieve natural pregnancy.

## Introduction

Several large-scale clinical trials have recently been performed to evaluate the safety and effectiveness of microbicidal vaginal gels in preventing HIV transmission [1], [2]. This prophylactic approach is favored in low-income and poorly educated populations due to its low cost and ease of use [3]. The development of topical microbicides with minimal impact on sperm function is especially important for HIV serodiscordant couples desiring pregnancy. Several reproductive options currently exist for these couples, including sperm washing and prophylactic antiretroviral therapy for the uninfected partner [4], [5]. Unfortunately, both of these options require access to medical care that may be difficult to achieve in certain high-risk populations. Consequently, the development of anti-HIV virucides that are not spermicidal is warranted. These compounds would address an unmet need in the global HIV community by providing an additional barrier to HIV infection in serodiscordant couples desiring pregnancy.

We recently developed a biocompatible cytolytic-peptide-loaded nanoparticle (NP) that inhibits HIV infectivity [6]. Melittin, the active agent, is a 26 amino acid amphipathic peptide component of bee venom. Free melittin rapidly integrates into lipid bilayers, oligomerizes and forms pores resulting in osmotic lysis [7]. The therapeutic utility of free melittin is limited by its cytotoxicity and propensity to induce hemolysis. However, melittin has also been shown to stably bind and insert into the unilayer lipid shell of NPs with an inert perfluorocarbon (PFC) core [8]. This process does not disrupt PFC NP structure and allows these NPs to serve as passively loaded drug delivery vehicles for melittin *in vivo*. In this way, non-specific melittin cytotoxicity is greatly reduced and no harmful effects are observed in mice even after multiple consecutive administrations of melittin NPs that exceed the known LD_50_ for free melittin [9].

Melittin NP (Mel-NP) membrane targeting can be made specific using ligands such as antibodies or peptidomimetics. Integrin-targeted mel-NPs have been shown to inhibit melanoma tumor growth *in vivo* [8], [9]. In the case of HIV, innate mel-NP fusogenic properties and electrostatic affinity for virions result in mel-NP mediated HIV capture and envelope disintegration [6]. Mel-NPs have been shown to inhibit HIV infectivity *in vitro* at equivalent melittin concentrations <10 µM (IC_50_ for CCR5-tropic virus: 3.6 µM) [6]. These NP concentrations were not cytotoxic to the mammalian reporter cells used in this assay.

Given these previous findings, the pre-clinical experiments herein test the hypothesis that packaging melittin into PFC NPs minimizes sperm and vaginal epithelial cell cytotoxicity. Addressing this hypothesis is the first step towards expanding the range of mel-NP applications to include use as a prophylactic vaginal virucide for HIV serodiscordant couples desiring pregnancy.

## Materials and Methods

### Ethics Statement

The collection and use of sperm for this study was approved by the Washington University School of Medicine Institutional Review Board. The Washington University Institutional review Board is responsible for ensuring ethics and patient protection in research at our institution. The IRB granted a waiver of patient consent given that we were using de-identified samples that were to be discarded. In all experiments, the samples were de-identified and the investigator performing the experiments was blinded to all patient identifiers.

### Nanoparticle Synthesis and Characterization

Perfluorocarbon nanoparticles (PFC NPs) were synthesized as previously described [10]. Briefly, a lipid surfactant co-mixture of 98.9 mol% egg lecithin, 0.1 mol% DiI and 1 mol% Carboxy-PEG-DSPE (Avanti Polar Lipids, Piscataway, NJ) was dissolved in chloroform, evaporated under reduced pressure and dried in a 50°C vacuum oven. DiI is a lipophilic carbocyanine dye commonly used for membrane labeling due to its strong fluorescence in hydrophobic environments and excellent retention in lipid membranes [11], [12]. The resulting lipid film (2.0% w/v) was resuspended in distilled deionized water and combined with perfluorooctylbromide (Gateway Specialty Chemicals, St. Peters, MO) (20% w/v) in a Tissumizer Mark II tissue homogenizer (Tekmar Company, Cincinnati, OH). This mixture was then continuously processed at 20,000 lbf/in^2^ for 4 min with an S110 Microfluidics emulsifier (Microfluidics, Newton, MA) to obtain an emulsion of PFC NPs. Subsequently, 25 ml of the emulsion was mixed with 1.95 mg avidin (Sigma-Aldrich, St. Louis, MO) for 15 min. Next, 7.163 mg ethylcarbodiimide hydrochloride (EDCI, Sigma- Aldrich, St. Louis, MO) was added for 40 min to facilitate covalent linkage between avidin and carboxy-PEG-DSPE on the NPs. Avidin NPs (blank NPs) were thus generated and dialyzed 3× in 2L of PBS for 30 min, overnight, and 30 min. NP size and zeta potential were determined using a ZetaPlus Zeta Potential analyzer (Brookhaven Instruments Corp., Holtsville, NY) and NPs were stored in phosphate-buffered saline at 4°C until use. When needed, the pH of phosphate-buffered saline was adjusted using hydrochloric acid.

The avidin (blank) NPs were the precursor to the anti-sperm NPs. To target sperm, anti-sperm adhesion molecule 1 (anti-SPAM1) antibodies (Santa Cruz Biotechnology, Santa Cruz, CA) were biotinylated and complexed with avidin NPs. To biotinylate the monoclonal anti-SPAM1 antibody, 0.2 mg/ml antibody was mixed with 8.24 µL 1 mM Biotin-NHS (Thermo Scientific, Rockford, IL) and incubated at room temperature for 30 minutes. To generate anti-SPAM1 NPs, 1.0 ml avidin NPs were mixed with 34.8 µL biotinylated antibody.

To load NPs with melittin, 1.0 mL of blank NPs or anti-SPAM1 NPs was incubated at a concentration of 1 mM melittin in water with rotation at 4°C for 72 hours. NPs were isolated by low speed centrifugation for 20 min. at 1000 g to “softly” pellet the NPs and were washed three times with phosphate-buffered saline. Control blank and anti-SPAM1 NPs underwent the same loading and washing protocols without the addition of melittin. Thus, the classes of NPs formed were: blank NPs, melittin NPs (mel-NPs), anti-SPAM1 NPs, and anti-SPAM1 melittin NPs (anti-SPAM1-mel-NPs).

The concentration of unbound melittin in the supernatants generated by this loading procedure was determined by reversed-phase high performance liquid chromatography (HPLC) using a Waters HPLC system (Waters Corporation, Milford, MA) and a Vydac 218TP54 (C18) column (Discovery Sciences, Albany, OR). The mobile phase consisted of a mixture of 0.1% trifluoroacetic acid (TFA) in water (solvent A) and 0.1% TFA in acetonitrile (solvent B). Composition of the mobile phase was varied from 60% solvent A/40% solvent B to 40% solvent A/60% solvent B over the course of 20 minutes and eluting peptides were detected by absorbance at 215 nm. Sample melittin concentrations were determined by comparing the area under the eluting melittin peak (retention time ∼8.2 minutes) to a standard curve generating using melittin samples of known concentration. The extent of melittin loading on NPs was determined by subtracting the amount of unbound melittin from the total amount of melittin originally added to the NPs. For assays involving both blank and melittin-loaded NPs, the “equivalent melittin concentration” of melittin-loaded NPs was calculated as described above and an equal number of blank NPs was used for comparison.

### Vaginal Epithelium Viability

Immortalized vaginal epithelial cells (VK2/E6E7) were obtained from ATCC (CRL-2616) and propagated according to the suggested protocol. For cytotoxicity studies, 15,000 cells were added to each well of a 96-well plate and allowed to attach for 24 hours. For single-dose studies, the agent of interest was then added and incubated with the cells for 12 hours at 37°C. This incubation time was chosen based on a tenofovir vaginal gel trial in which participants were instructed to apply the gel twice in the 24-hour period surrounding sexual activity [1]. In repeat dosing studies, the media was replaced by fresh media containing the agent of interest once per day for 3 days. Following incubation, cells were washed once with media and incubated with the MTT reagent (3-(4,5-dimethylthiazol-2-yl)-2,5-diphenyl tetrasodium bromide) for 4 hours. The colored product was solubilized in DMSO and absorbance at 570 nm was measured using a plate reader.

### Nanoparticle-Sperm Interactions

Human semen samples were obtained from the Washington University in St. Louis *in vitro* fertilization laboratory and stored at 37°C until use. Each treatment group (blank NPs, anti-SPAM1 NPs) consisted of samples from three different sperm donors. Thus, a total of six fresh semen samples were used to assess the interaction of NPs with sperm. Motile sperm were isolated from each sample by density gradient centrifugation using a discontinuous Percoll gradient [13]. Briefly, 100% isotonic Percoll solution was generated by mixing Percoll (Sigma-Aldrich, St. Louis, MO) with 10× phosphate-buffered saline at a volume ratio of 9∶1. Further dilutions of this solution were made using 1× phosphate-buffered saline. A centrifuge tube containing layers of 80% isotonic Percoll, 40% isotonic Percoll, and semen was carefully prepared and centrifuged at 1000 g for 20 minutes to pellet motile sperm. Afterwards, the supernatant was removed and the pelleted sperm were resuspended in 4% paraformaldehyde and fixed at 37°C for 30 minutes. Fixed sperm were then washed three times with phosphate-buffered saline to remove residual paraformaldehyde.

To determine the extent of NP binding to sperm, fluorescent NPs at the appropriate concentration were added to sperm to achieve a final sperm concentration of 70 million per mL. For comparison with the results of other assays, the highest number of NPs added in this assay (3×10^10^ NPs) corresponded to the highest NP concentration tested in the motility and viability assays (80 µM equivalent melittin concentration). Samples were incubated for 30 minutes at 37°C and were pipetted up and down 3 times every 10 minutes to keep the sperm and NPs suspended. Afterwards, the sperm/NP mixture was layered onto 100% isotonic Optiprep medium (density = 1.32 g/mL, Sigma-Aldrich, St. Louis, MO) and centrifuged at 1000 g for 20 minutes. Due to the high density of the PFC NPs (∼1.9 g/mL [14]), unbound NPs formed a pellet while sperm and their associated NPs formed a band above the Optiprep layer. This band was collected and NP content was determined by measuring fluorescence at an excitation wavelength of 549 nm and an emission wavelength of 565 nm. These measurements were obtained using a Cary Eclipse spectrophotometer (Varian, Inc., Walnut Creek, CA) and compared to a standard curve generated using NP solutions of known concentration. Control runs containing NPs and no sperm were also performed to quantify background fluorescence due to unbound NPs that failed to pellet during the centrifugation.

### Sperm Aggregation

Human semen samples were obtained from the Washington University in St. Louis *in vitro* fertilization laboratory and stored at 37°C until use. A single fresh semen sample was used for the aggregation experiments. Semen was diluted in EmbryoMax Human Tubal Fluid (HTF, Millipore) and 3×10^10^ fluorescent NPs were added to achieve a final sperm concentration of 70 million per mL. Samples were incubated for 30 minutes at 37°C and were pipetted up and down 3 times every 10 minutes to keep the sperm and NPs suspended. Afterwards, sperm aggregation was visualized by brightfield and fluorescent microscopy.

### Sperm Motility and Viability

Human semen samples were obtained from the Washington University in St. Louis *in vitro* fertilization laboratory and stored at 37°C until use. Each treatment group (free melittin, blank NPs, mel-NPs, anti-SPAM1 NPs and anti-SPAM1 mel-NPs) consisted of samples from three different sperm donors. Thus, a total of fifteen fresh semen samples were used for motility and viability experiments. All semen samples used in this study had >50% motility at the time of donation and were used on the day of donation. Semen was diluted in EmbryoMax Human Tubal Fluid (HTF, Millipore, Billerica, MA), aliquoted, and the agent of interest was added to achieve a final sperm concentration of 10 million per mL. Samples were incubated for 30 minutes at 37°C and were pipetted up and down 3 times every 10 minutes to keep the sperm and NPs suspended. This incubation time was chosen based on previous reports indicating that sperm leave the vagina within minutes of deposition and are found throughout the female reproductive tract 30 minutes after insemination [15], [16]. Afterwards, VIADENT stain (Hoechst 33258, Hamilton Thorne, Beverly, MA) was added to achieve a final stain concentration of 10 µg/mL and samples were incubated for an additional 5 minutes. This stain is membrane-impermeant and only labels nonviable cells with disrupted membrane integrity. Percent motility and viability were determined using an IVOS Computer Assisted Sperm Analyzer (Hamilton Thorne, Beverly, MA). The motion of each sperm head identified in the sample was recorded for one second and used to generate a smoothed trajectory curve. A spermatozoon was considered to be motile if its average velocity along this path exceeded 10 µm/second. A spermatozoon was considered to be viable if it did not demonstrate detectable nuclear staining following addition of the VIADENT stain. Motility and viability measurements following treatment were then normalized to the motility and viability of untreated sperm from each semen sample.

### Statistical Analysis

For simple two-sample comparisons, an unpaired Student's t analysis was performed assuming a normal sample distribution and α = 0.05. For comparisons of more than two samples, a one-way analysis of variance (ANOVA) followed by Tukey HSD analysis of all possible sample pairings was performed using JMP Version 10 (SAS Institute, Cary, NC) statistical software assuming a normal sample distribution and α = 0.05.

## Results

### Melittin Loading and Nanoparticle Stability

**Figure 1:**
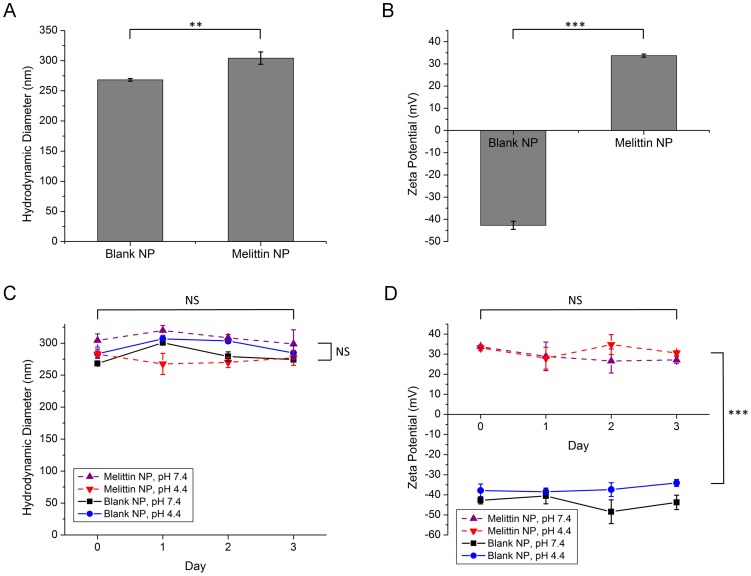
Physicochemical properties and stability of blank NPs and mel-NPs. (**A** and **B**) Size and zeta potential of blank NPs and mel-NPs immediately following preparation. (**C** and **D**) Size and zeta potential of blank NPs and mel-NPs following incubation for up to 3 days at pH 7.4 or pH 4.4. Error bars represent S.D. of n = 3 replicates, NS p>0.05, ** p<0.01, *** p<0.001.

In order to characterize the loading of melittin onto perfluorocarbon nanoparticles (PFC NPs), we compared the size and zeta potential of blank NPs to those of mel-NPs immediately following preparation. Blank NPs were found to be 268 nm in diameter while mel-NPs were 304 nm in diameter, indicating a slight but statistically significant increase in size following melittin loading (**Fig. 1A**, p = 0.004). In contrast, the zeta potential of blank NPs was −42.7 mV while the zeta potential of mel-NPs was 33.7 mV (**Fig. 1B**, p<0.0001). These results demonstrate a dramatic reversal of zeta potential upon loading of the cationic melittin peptide. HPLC analysis was performed to further quantify the extent of melittin loading and indicated a loading efficiency of 41.9% with an estimated 16,210 melittin peptides per NP.

NP stability was assessed by monitoring the size and zeta potential of NPs stored at either pH 7.4 or pH 4.4. Storage at pH 7.4 was used to determine NP stability in phosphate-buffered saline and many biological environments, including plasma and cellular media. In contrast, storage at pH 4.4 was used to assess NP stability in the acidic vaginal environment. No significant changes in size or zeta potential were observed over the course of three days in any of the storage conditions (**Figs. 1C and 1D**). Importantly, mel-NPs stored at pH 4.4 for three days did not exhibit any significant difference in size (p = 0.48) or zeta potential (p = 0.08) compared to freshly prepared mel-NPs. After three days of storage at pH 4.4, mel-NPs had a zeta potential of 30.6 mV, which was substantially more positive than that of blank NPs stored under the same conditions (−34.1 mV, p<0.0001).

### Vaginal Epithelium Toxicity

**Figure 2:**
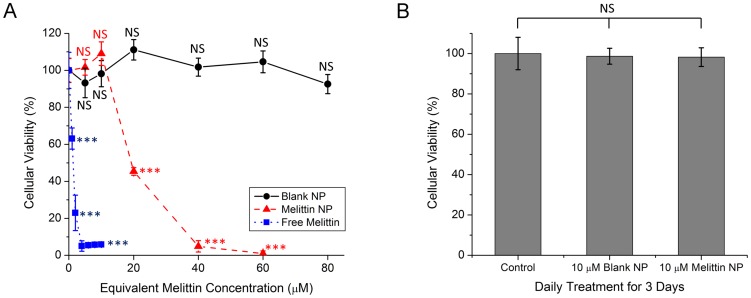
Effect of free melittin, blank NPs and mel-NPs on vaginal epithelial cell viability. (**A**) VK2 vaginal cell viability following treatment with a single dose of free melittin, blank NPs or mel-NPs for 12 hours. (**B**) VK2 vaginal cell viability following repeated treatment with fresh media (control) or a 10 µM equivalent melittin dose of blank NPs or mel-NPs once per day for 3 days. VK2 viability was determined using MTT assay. Error bars represent S.D. of n = 6 replicates. Labels indicate level of statistical significance compared to untreated control, NS p>0.05, *** p<0.001.

In order to determine the impact of NP incorporation on the cytotoxicity of melittin, we assessed vaginal epithelial cell viability following exposure to free melittin, mel-NPs, or blank NPs for 12 hours (**Fig. 2A**). Vaginal cell viability was significantly reduced upon exposure to free melittin at concentrations ≥1 µM (p<0.001 relative to control). The IC_50_ for cell viability was 1.3 µM and exposure to free melittin at concentrations ≥4 µM resulted in essentially complete loss of vaginal cell viability. In contrast, mel-NPs did not significantly affect vaginal cell viability at equivalent melittin concentrations up to 10 µM (p = 0.48) and exhibited an IC_50_ for cell viability of 24.3 µM. At equivalent melittin concentrations ≥40 µM, mel-NPs also resulted in essentially complete loss of vaginal cell viability. Blank NPs did not significantly affect vaginal cell viability at any concentration tested up to 80 µM equivalent melittin concentration (p = 0.81).

Given that a single treatment with mel-NPs at an equivalent melittin concentration of 10 µM did not significantly affect vaginal cell viability, we next tested whether multiple treatments of blank NPs or mel-NPs at this concentration would be cytotoxic (**Fig. 2B**). There was no significant effect on cell viability following daily treatment for three days with either blank NPs or mel-NPs at 10 µM equivalent melittin concentration (p = 0.86).

### Sperm Targeting

**Figure 3:**
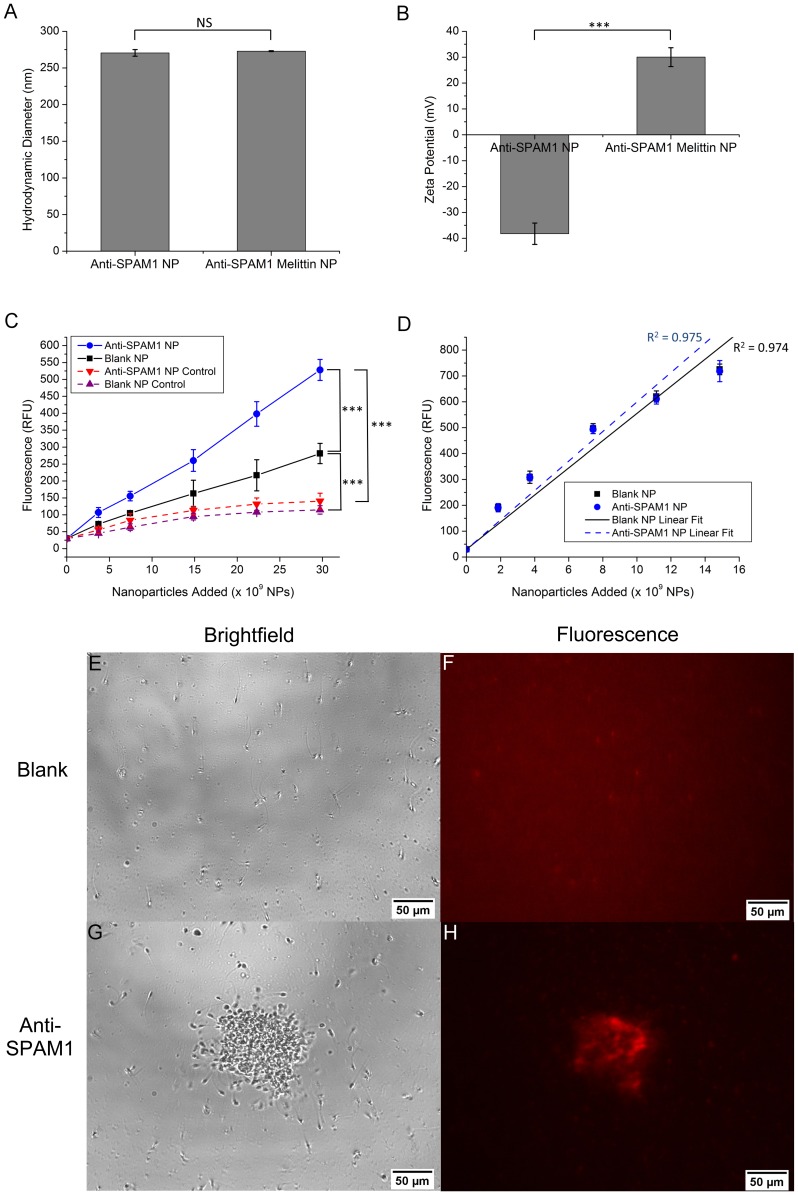
Characterization of SPAM1-targeted NPs and their interactions with sperm. (**A** and **B**) Size and zeta potential of anti-SPAM1 NPs and anti-SPAM1-mel-NPs immediately following preparation. (**C**) Fluorescence of sperm-bound blank NPs and anti-SPAM1 NPs following incubation with sperm for 30 minutes and removal of unbound NPs by density gradient centrifugation. Control samples did not contain sperm and were used to determine background fluorescence due to remaining unbound NPs. (**D**) Standard curve of blank NP and anti-SPAM1 NP fluorescence used for quantification of sperm binding. Error bars represent S.D. of n = 3 replicates, NS p>0.05, *** p<0.001. (**E**, **F**, **G** and **H**) Brightfield and fluorescence images of semen samples following addition of 3×10^10^ blank NPs or anti-SPAM1 NPs. Scale bar = 50 µm.

For comparison with untargeted NPs in terms of sperm affinity and toxicity, we next conjugated an antibody to sperm adhesion molecule 1 (SPAM1) to the surface of our blank NPs. The SPAM1 hyaluronidase is a highly conserved sperm surface antigen that has previously been used as a target for immunocontraception [17], [18]. We reasoned that anti-SPAM1 targeting would maximize interactions between NPs and sperm, thus revealing the upper limit of NP cytotoxic potential. Targeted NPs were similar to untargeted NPs in terms of size and zeta potential (**Figs. 3A and 3B**). Anti-SPAM1 NPs had a diameter of 271 nm and zeta potential of −38.2 mV, while anti-SPAM1-mel-NPs had a diameter of 273 nm and zeta potential of 30.0 mV. Thus, melittin loading onto targeted NPs did not significantly impact NP size (p = 0.42), but caused a dramatic reversal of zeta potential (p<0.0001). To examine NP-sperm interactions, fluorescent NPs were added to sperm for 30 minutes, followed by density gradient centrifugation to separate sperm-bound NPs from unbound NPs. The extent of NP binding to sperm was then quantified using fluorescence spectrophotometry (**Fig. 3C**). Both blank and anti-SPAM1 NPs exhibited bound NP fluorescence greater than background control at all concentrations tested (p<0.001 for addition of 3×10^10^ NPs). However, anti-SPAM1 NPs resulted in significantly higher fluorescence than blank NPs at all concentrations tested (p<0.001 for addition of 3×10^10^ NPs). Comparison of measured fluorescence values with a standard curve (**Fig. 3D**) indicated that addition of 3×10^10^ NPs resulted in binding of approximately 372 blank NPs and 898 anti-SPAM1 NPs to each spermatozoon. Interestingly, when 3×10^10^ NPs were added to fresh semen samples, blank NPs produced a uniform distribution of individual motile sperm (**Fig. 3E**) and diffuse NP fluorescence (**Fig. 3F**), while anti-SPAM1 NPs produced large fluorescent complexes containing NPs and visibly intact sperm with hindered motility (**Figs. 3G and 3H**).

### Sperm Toxicity

**Figure 4:**
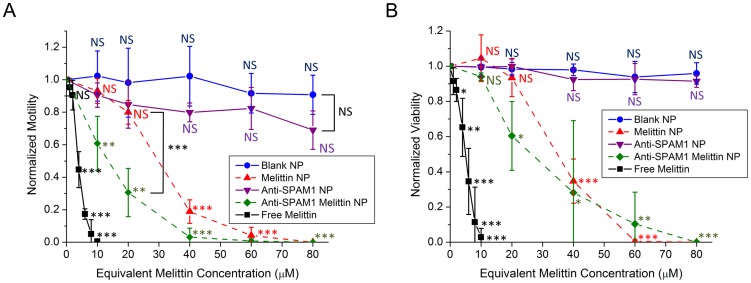
Effect of free melittin, blank NPs, mel-NPs, anti-SPAM1 NPs, and anti-SPAM1-mel-NPs on sperm motility and viability. (A and B) Sperm motility and viability following treatment with the appropriate agent for 30 minutes. Motility and viability were determined by IVOS Computer Assisted Sperm Analysis and normalized to the motility and viability of untreated sperm. Error bars represent S.D. of n = 3 replicates using sperm samples from distinct donors. Labels indicate level of statistical significance compared to untreated control, NS p>0.05, * p<0.05, ** p<0.01, *** p<0.001.

Finally, we compared the effects of free melittin, blank NPs, mel-NPs, anti-SPAM1 NPs, and anti-SPAM1-mel-NPs on sperm motility (**Fig. 4A**) and viability (**Fig. 4B**). Both motility and viability were significantly reduced following exposure to >2 µM free melittin (p<0.001 relative to control). The IC_50_'s for sperm motility and viability were 4.0 µM and 4.9 µM respectively. Exposure to ≥8 µM free melittin resulted in essentially complete elimination of sperm motility and viability. In contrast, intragroup analysis indicated substantially lower toxicity for melittin incorporated into NPs. Significant loss of motility only occurred following exposure to ≥40 µM of mel-NPs (p<0.001) or ≥10 µM of anti-SPAM1-mel-NPs (p = 0.003). Significant loss of viability occurred following exposure to ≥40 µM of mel-NPs (p<0.001) or ≥20 µM of anti-SPAM1-mel-NPs (p = 0.03). The IC_50_'s for sperm motility were 28.8 µM for mel-NPs and 14.0 µM for anti-SPAM1-mel-NPs, while the IC_50_'s for sperm viability were 36.9 µM for mel-NPs and 28.5 µM for anti-SPAM1-mel-NPs. Exposure to 80 µM equivalent melittin concentration of either mel-NPs or anti-SPAM1-mel-NPs resulted in complete loss of motility and viability. Blank NPs and anti-SPAM1 NPs which did not contain melittin were substantially less toxic. Blank NPs had no significant effect on sperm motility (p = 1.0) or viability (p = 0.42) even at the highest concentration tested and anti-SPAM1 NPs only caused a slight reduction in sperm motility to 69.0% at this concentration (p = 0.06). Intergroup analysis indicated that anti-SPAM1-mel-NPs were more toxic than untargeted mel-NPs at intermediate concentrations such as 20 µM (p<0.001). Overall, both mel-NPs and anti-SPAM1-mel-NPs were more toxic than NPs that did not contain melittin (p<0.001 at 80 µM equivalent melittin concentration) and substantially less toxic than free melittin (p<0.001 at 10 µM equivalent melittin concentration).

## Discussion

These data confirm our hypothesis that formulation of melittin into NPs significantly decreases sperm and vaginal epithelial cell cytotoxicity. Free melittin reduces both sperm and vaginal epithelial cell viability at concentrations >2 µM while mel-NP cytotoxicity requires an equivalent melittin concentration ≥20 µM. This difference is best explained by the NP capacity for reducing melittin activity, combined with the presence of polyethylene glycol (PEG) on the NP surface. Previous studies support our results in that PEGylation of NPs prevents their interaction and uptake by vaginal and mononuclear cells making them safe for use as topical vaginal microbicides [19]. Importantly, blank NPs did not affect cell viability at any concentration tested, indicating that mel-NP cytotoxicity is due to melittin delivery and not inherent toxicity of the NP carrier.

Size and zeta potential are two key parameters which influence NP behavior in biological systems. All NPs generated during this study were approximately 250-300 nm in diameter, consistent with those used in our previous study [6]. The relatively large size of these NPs may prevent penetration of the vaginal epithelial barrier, thereby minimizing systemic absorption of melittin following application [20]. Additionally, size and zeta potential analysis confirmed stability of both blank NPs and mel-NPs at vaginal pH for at least three days, further supporting the use of these NPs as intravaginal melittin delivery agents.

Our long-term goal is to develop a vaginal gel-based mel-NP platform for HIV serodiscordant couples. This will require additional safety and efficacy experiments in animal model systems more closely simulating the true vaginal environment. Our current results indicate that mel-NPs are not toxic to vaginal cells at equivalent melittin concentrations up to 20 µM. It is expected that gel suspension of NPs will further increase NP safety because the gel will attenuate contact between NPs and vaginal epithelium. Further studies will also be needed to characterize the dynamics of melittin delivery *in vivo* and determine the optimal time for gel application prior to sexual activity.

For optimal therapeutic utility in serodiscordant couples desiring pregnancy, the mel-NP concentration must be sufficient to completely inhibit HIV infectivity while simultaneously preventing sperm and vaginal toxicity. Based on these criteria, our results demonstrate that an equivalent melittin concentration of 10 µM could serve as a starting point for future investigation. However, this NP concentration may need to be adjusted based on the results of *in vivo* toxicity testing. Interestingly, mel-NP toxicity to sperm was enhanced with sperm targeting. Thus, the targeted mel-NP platform might be adapted for alternative use as a contraceptive agent.

The membranolytic activity of mel-NPs makes them highly effective virucides of enveloped viruses such as HIV. Rapid mel-NP mediated rupture is facilitated by the relatively small virion surface area, high internal pressure and inability to repair membrane defects [6]. It is unlikely that HIV will acquire mutational resistance against this virucidal mechanism. This approach would be capable of inactivating both free virions and those transmitted through sperm surface associations [21], [22].

HIV-infected seminal leukocytes also mediate infectivity [23]. While the current mel-NP design does not target leukocytes, our data demonstrate that anti-sperm targeted NPs could potentially aggregate infective sperm. It follows that a similar strategy could be employed to selectively target cluster of differentiation and/or HIV infectivity markers on the surface of infected seminal leukocytes. Such NPs might effectively prevent cell to cell “virological synapse” mediated HIV transmission through aggregation and lysis of infective seminal cells.

In conclusion, we have demonstrated the proof of concept that it is possible to design an HIV virucide with minimized cytotoxicity to sperm and vaginal epithelium. Mel-NPs can effectively serve as an additional barrier to HIV transmission that might one day find favorable use by serodiscordant couples desiring pregnancy.
